# Calcifying Epithelial Odontogenic Tumor: A Report of a Rare Case With Clinical and Radiographic Findings

**DOI:** 10.7759/cureus.113482

**Published:** 2026-07-27

**Authors:** Adam N Daou, Said Al Halabi, Ghassan Yared, Wassim Manhal

**Affiliations:** 1 Department of Oral Pathology, Faculty of Dental Medicine, Université Saint-Joseph de Beyrouth, Beirut, LBN; 2 Department of Oral and Maxillofacial Surgery, Private Practice, Beirut, LBN; 3 Department of Oral Medicine and Radiology, Faculty of Dental Medicine, Université Saint-Joseph de Beyrouth, Beirut, LBN

**Keywords:** calcifying epithelial odontogenic tumor, neoplasms, odontogenic tumors, oral medicine, oral pathology

## Abstract

This case report describes a calcifying epithelial odontogenic tumor (CEOT), also known as a Pindborg tumor, occurring in a 23-year-old male and located in the anterior mandible. Clinically, the patient presented with a significant swelling extending from the lower lip to the chin region. The swelling was firm and non-pitting on palpation. Following comprehensive radiographic evaluation and treatment planning, the lesion was managed by enucleation using a conservative surgical approach aimed at preserving as much bone as possible. The present case highlights the critical role of comprehensive clinical, radiographic, and histopathological evaluation in the accurate diagnosis and management of CEOT.

## Introduction

Calcifying epithelial odontogenic tumor (CEOT) is a benign epithelial odontogenic neoplasm derived from odontogenic epithelium [1]. It was first described by the Danish pathologist Jens J. Pindborg in 1955 and was subsequently reported in his landmark publication in 1958, from which the lesion derives its eponym, the Pindborg tumor [2]. CEOT is a slow-growing but locally invasive lesion and is considered relatively rare, accounting for less than 1% of all specimens submitted to oral pathology laboratories [3]. In the majority of cases, CEOT presents as an intraosseous lesion, while extraosseous (peripheral) variants are uncommon, representing fewer than 5% of cases [4].

The histogenesis of CEOT remains controversial. Some authors propose that the tumor originates from the stratum intermedium of the enamel organ during odontogenesis, whereas others suggest derivation from residual cells of the dental lamina present in the early stages of tooth development [5].

The aim of this case report is to detail the diagnostic approach to the lesion and to describe a surgical technique designed to preserve the maximum amount of healthy bone.

## Case presentation

A 23-year-old Lebanese male of Middle Eastern ethnicity presented to our clinic after referral from a fellow dentist with the chief complaint of swelling in the anterior region of the mandible. The patient reported no associated pain. His medical history was non-contributory, with no known systemic diseases, regular medication use, allergies, or previous maxillofacial pathology. Family history was unremarkable for odontogenic tumors or other relevant hereditary conditions.

**Figure 1:**
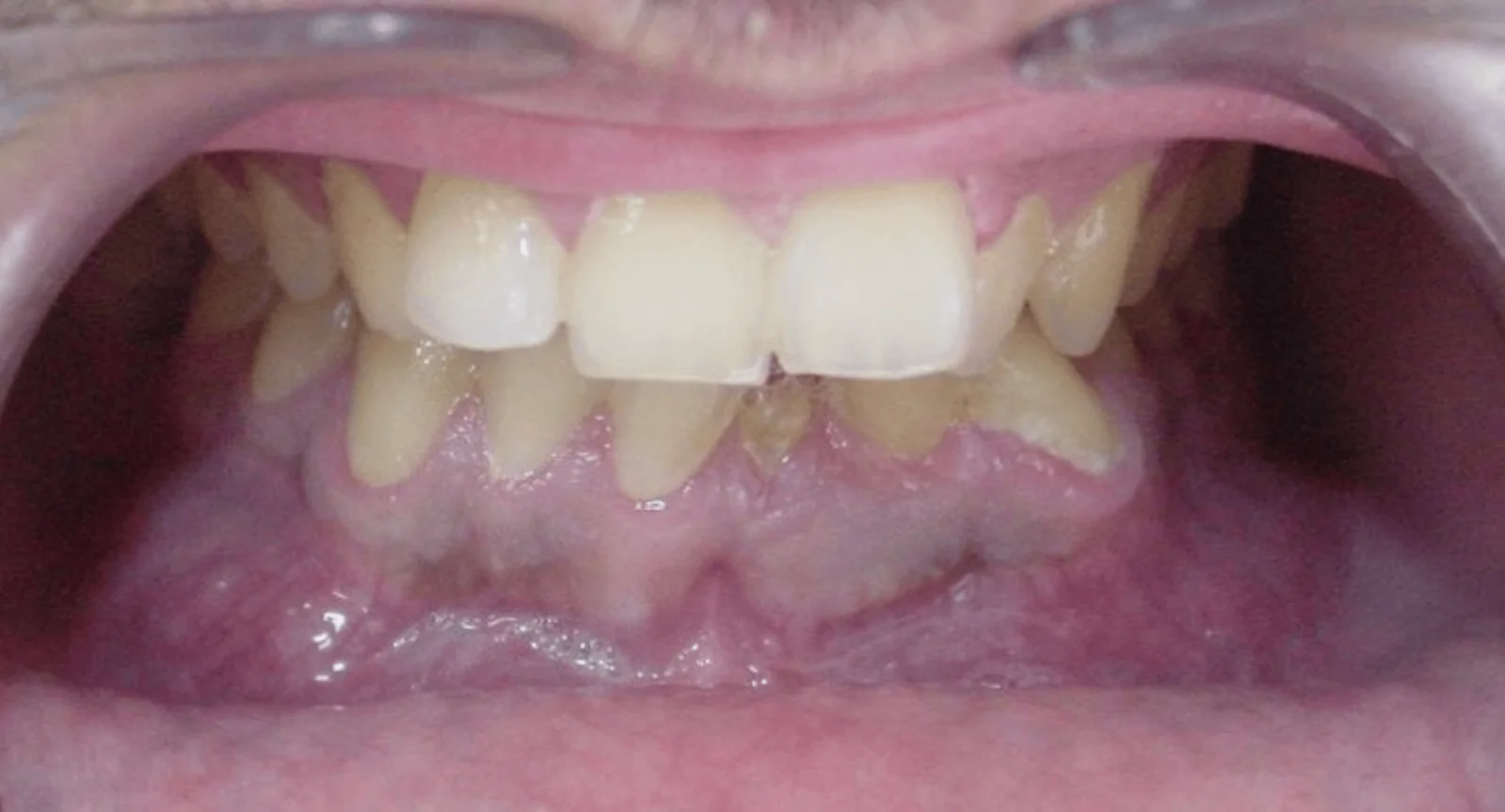
Image of the overlying mucosa on day 0 upon presenting to the clinic

Extraoral examination revealed a significant swelling extending between the lower lip and the chin. The swelling was non-pitting and hard on touch. In addition, non-tender and palpable submandibular lymph nodes were present. Intraoral examination demonstrated a swelling that was firm on palpation, measuring approximately 6 × 3 cm in the anterior mandibular region. The overlying mucosa appeared normal with no evidence of ulceration (Figure 1).

Palpation revealed expansion and tenderness along the inferior border of the mandible in the region of the lesion. No fistula formation or purulent discharge was observed on either intraoral or extraoral examination. The patient did not report symptoms of dysphagia or dyspnea. Pulp vitality testing, performed using both cold and electric pulp tests, indicated that the teeth adjacent to the lesion were vital.

**Figure 2:**
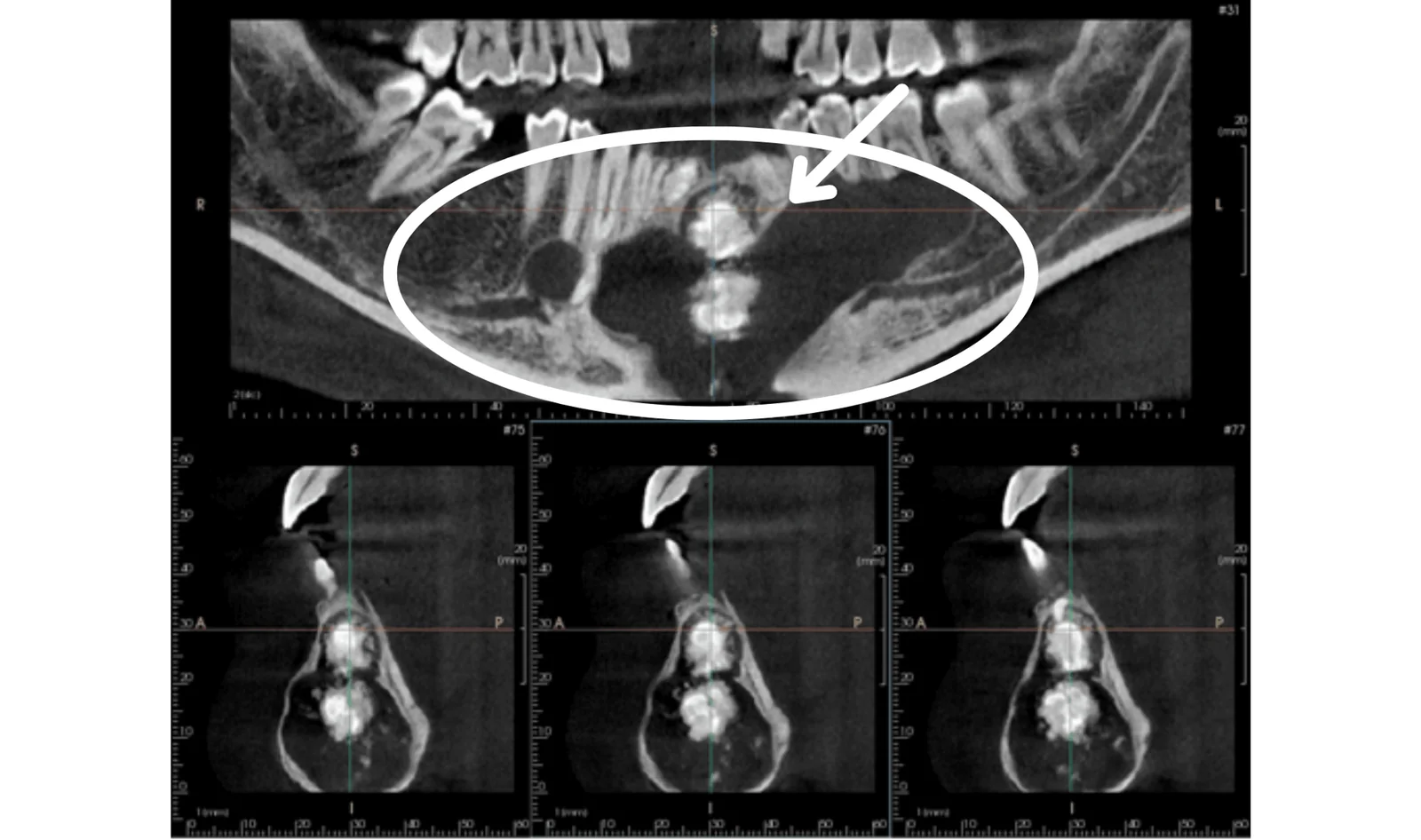
CBCT imaging illustrating the full extent of the lesion and delineating the associated radiopaque structures CBCT: cone-beam computed tomography

Panoramic radiography demonstrated a well-circumscribed mixed radiolucent-radiopaque lesion with corticated borders located in the anterior mandible. To obtain more precise measurements and accurately evaluate the size of the lesion, a cone-beam computed tomography (CBCT) scan was requested, as it provides high-resolution three-dimensional imaging of the maxillofacial structures (Figure 2) [6]. The lesion extended from the right mandibular second premolar to the distal root of the left mandibular first molar, with mixed radiopaque and radiolucent findings, resulting in significant displacement of the adjacent tooth roots. Inferior extension toward the lower border of the mandible was also noted. The highlighted cross-sectional image demonstrates the presence of intralesional calcifications, with no evidence of perforation of either the buccal or lingual cortical plates (Figure 2).

**Figure 3:**
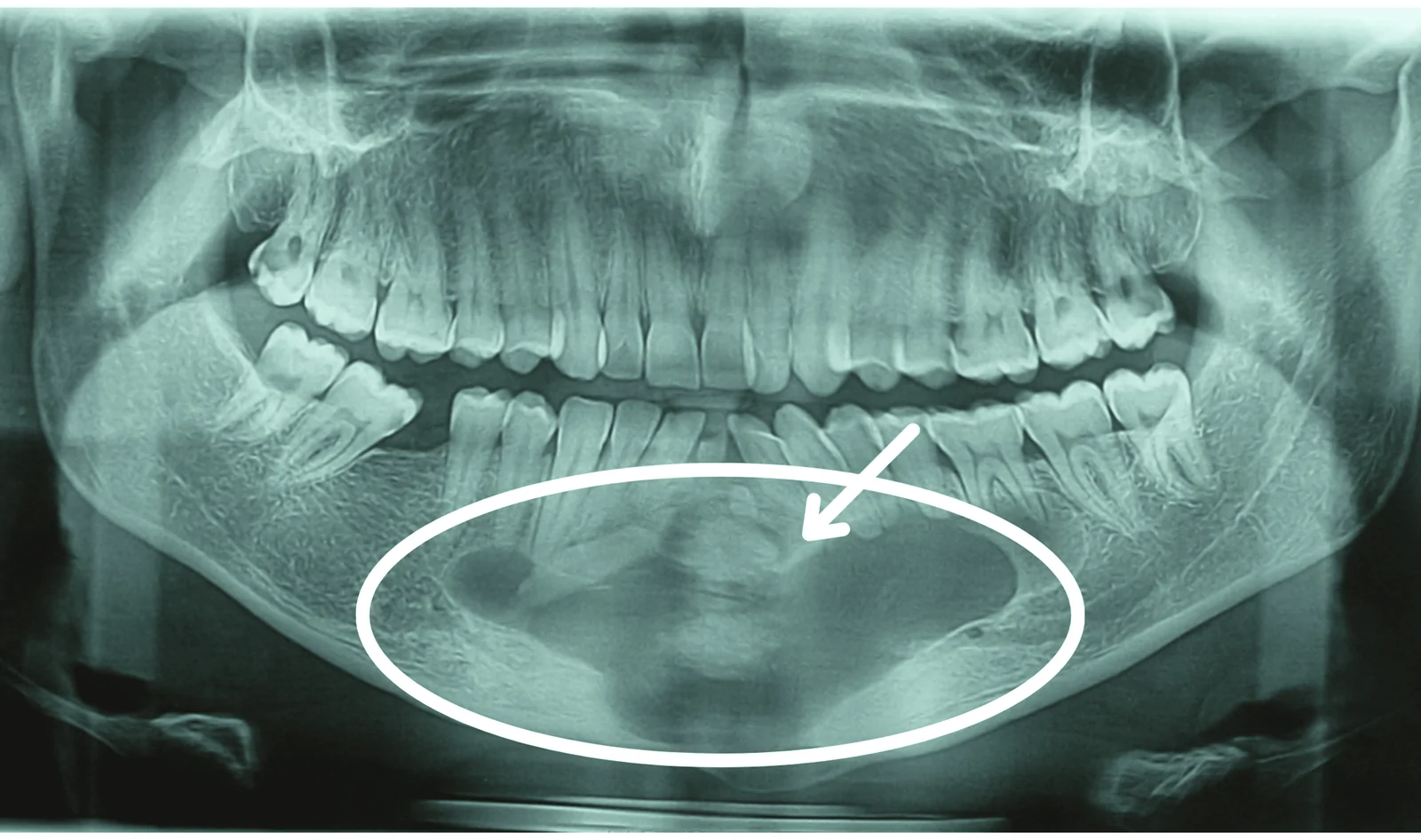
Panoramic X-ray of the patient on day 0

Two radiopaque, odontoma-like structures were identified within the central portion of the lesion. In addition, two supernumerary teeth were observed superior to these radiopaque masses. Notably, tooth #31 was visible inferior to the roots of teeth #41 through #43 on the panoramic radiograph (Figure 3), although it was not obstructed in eruption by the lesion and appeared to be displaced.

The patient underwent surgical enucleation of the lesion under general anesthesia and was placed in the supine position with nasotracheal intubation. A full-thickness mucoperiosteal flap was raised through a sulcular incision extending from the right second molar to the left second molar, with bilateral vertical releasing incisions to facilitate surgical access. Following subperiosteal dissection, careful identification and mobilization of the mental nerve from the surrounding periosteum were performed to prevent nerve injury during flap elevation.

After flap reflection, areas of buccal cortical plate perforation were observed, likely secondary to pressure exerted by the lesion. However, the existing osseous windows were insufficient to allow complete detachment of the lesion from the surrounding bone. Therefore, additional access windows were conservatively created in regions where the cortical bone was markedly thinned and fragile, with the aim of preserving as much intact bone as possible.

**Figure 4:**
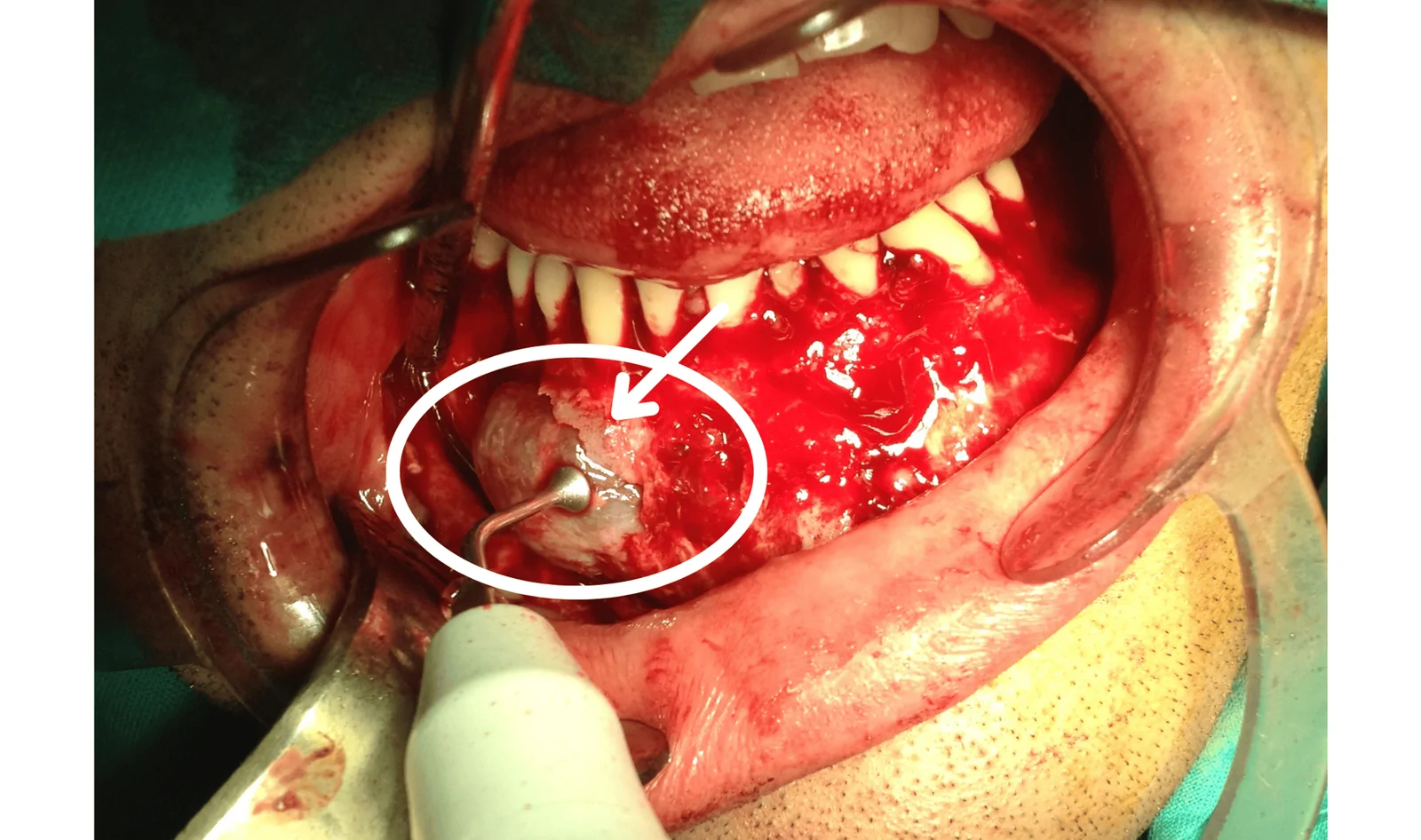
Piezoelectric surgical device with a SL3 bur used to detach the lesion from the surrounding bone

The Acteon Piezotome 2 (Acteon Group (Satelec), Mérignac, France) was equipped with an SL3 tip and utilized to facilitate atraumatic separation of the lesion from the surrounding cortical bone (Figure 4). This is achievable because piezo technology facilitates micrometric cuts for microsurgery while preserving maximum accuracy [7]. The SL3 tip, a non-cutting, plateau-shaped insert primarily designed for sinus membrane elevation, was selected for its tissue-selective properties, allowing careful detachment of the lesion from the bone while minimizing the risk of perforation or rupture.

**Figure 5:**
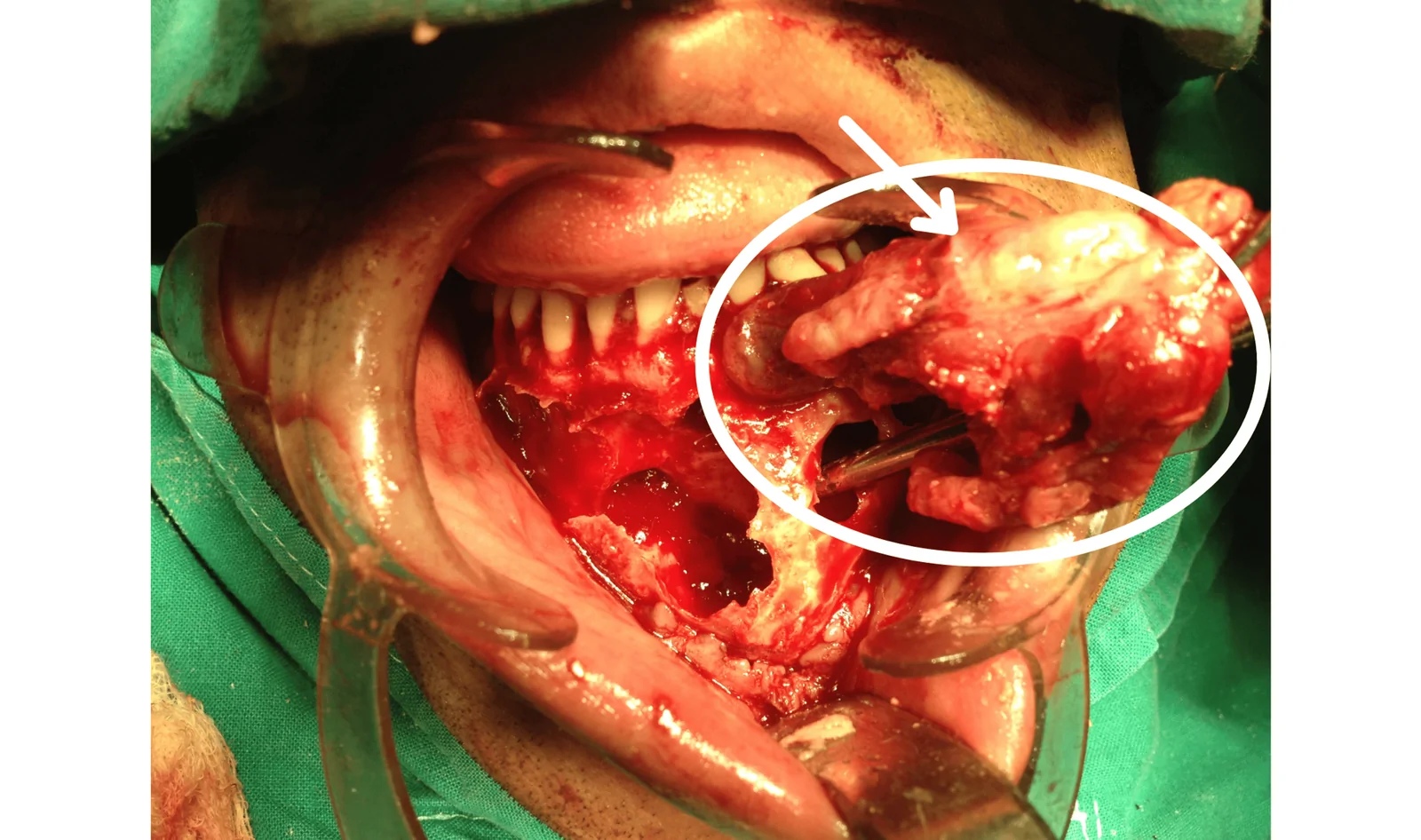
Intraoperative view during enucleation of the lesion

**Figure 6:**
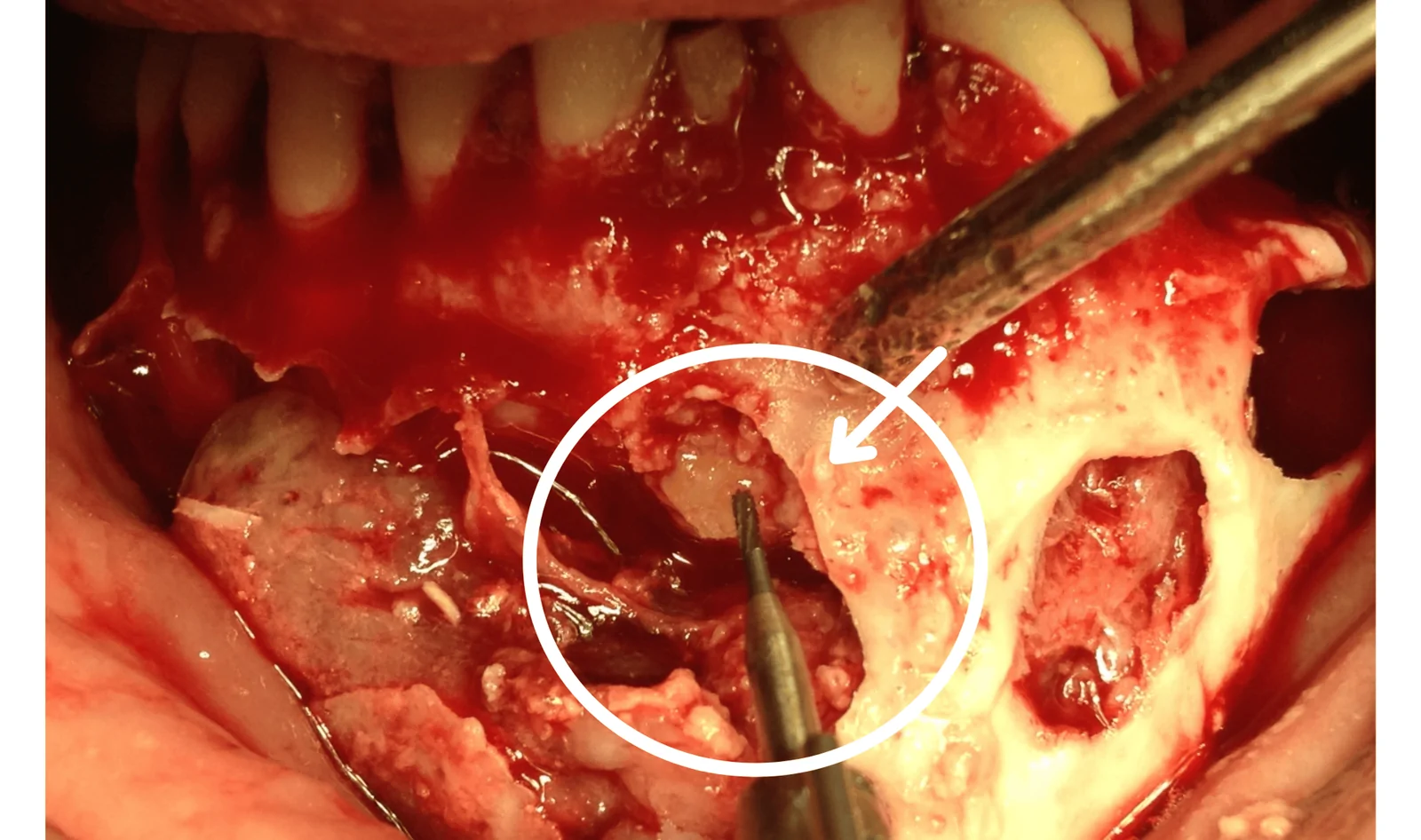
Fragmentation of the calcified structures using a cylindrical bur

Following enucleation of the lesion (Figure 5), access to tooth #31 was achieved, allowing for its extraction. The removal of the calcified structures, however, proved to be more challenging. In accordance with the initial objective of preserving as much bone as possible, the calcifications were carefully sectioned using a straight cylindrical bur mounted on a handpiece to facilitate their removal (Figure 6).

**Figure 7:**
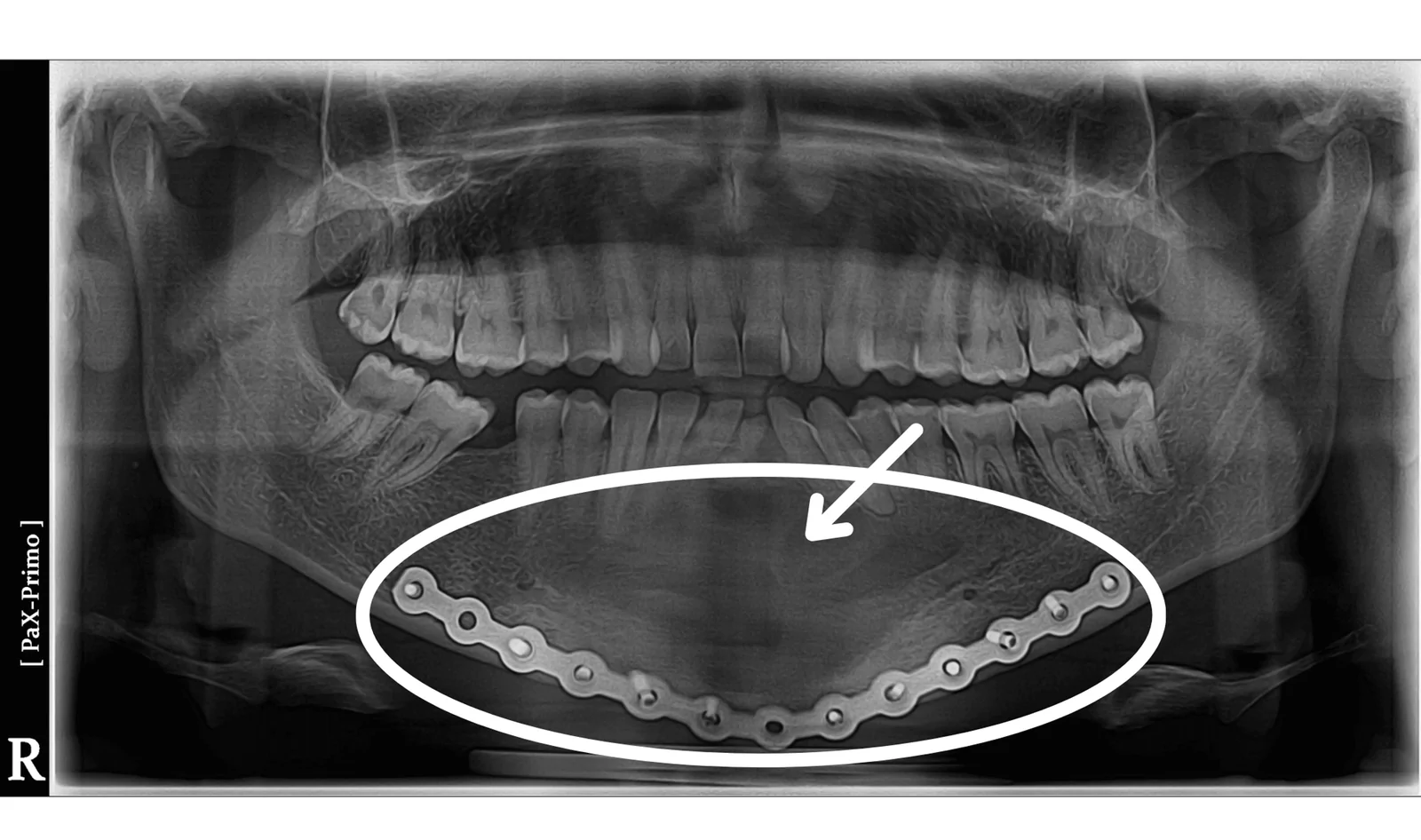
Post-op panoramic X-ray

In addition, a round bur mounted on a handpiece was used to perform bone remodeling. Following complete removal of the calcified structures and finalization of osseous contouring, a load-bearing 2.6 mm mandibular reconstruction plate (Anthon Hipp., Tuttlingen, Germany) was placed on the inferior border of the mandible under the mental foramen and secured with 11 2.4 mm fixation screws (Anthon Hipp., Tuttlingen, Germany) to reinforce mandibular integrity and facilitate proper healing (Figure 7).

**Figure 8:**
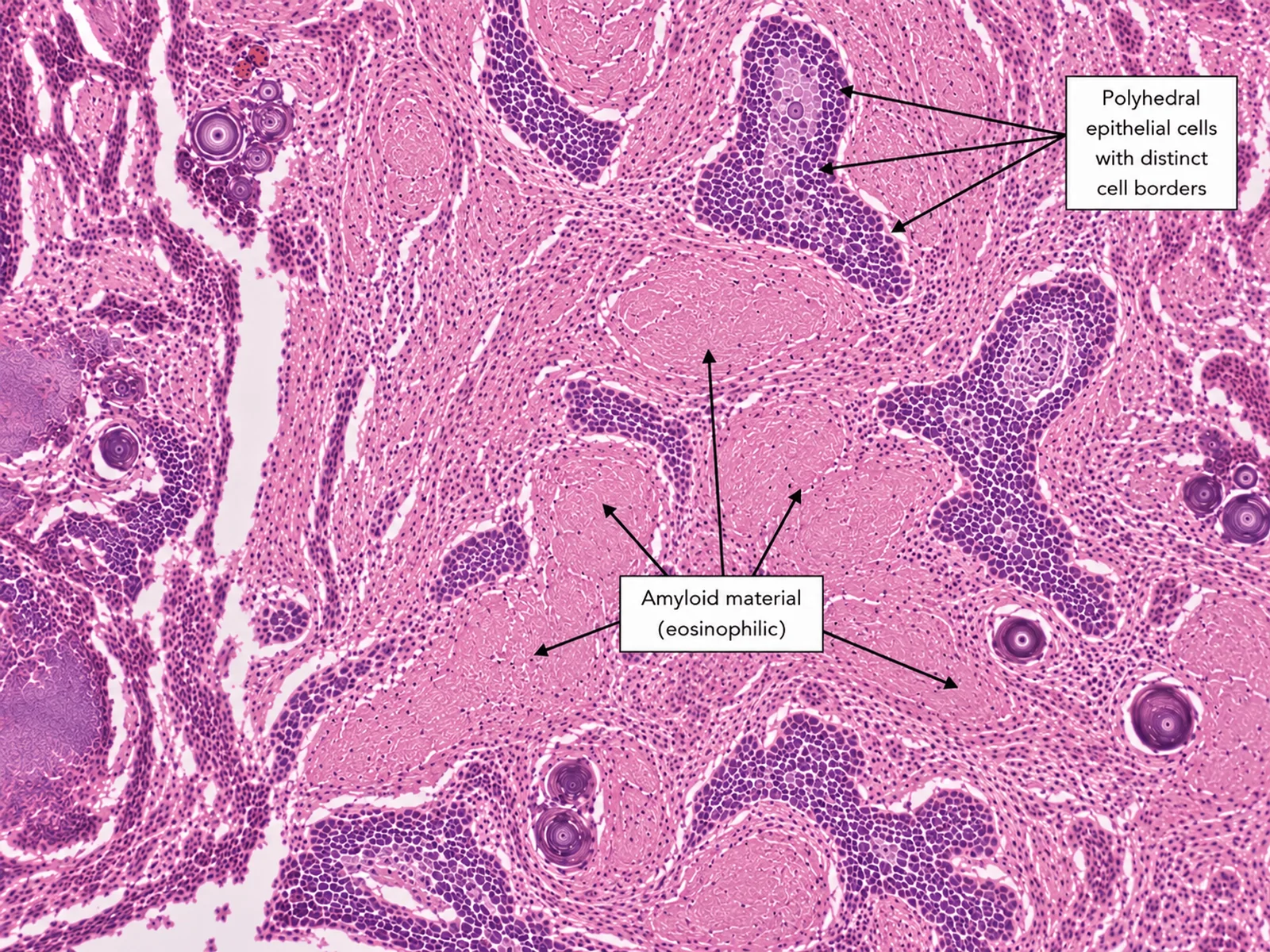
Calcifying epithelial odontogenic tumor (Pindborg tumor); photomicrograph showing islands and cords of polyhedral epithelial cells with distinct cell borders embedded in abundant eosinophilic amyloid-like material (hematoxylin and eosin stain)

The excised specimen was subsequently submitted for histopathological examination, which established the diagnosis of a CEOT associated with an adjacent odontoma (Figure 8). It is noteworthy that at the three-month follow-up, pulp vitality tests were repeated, and all involved teeth remained vital and intact. 

**Figure 9:**
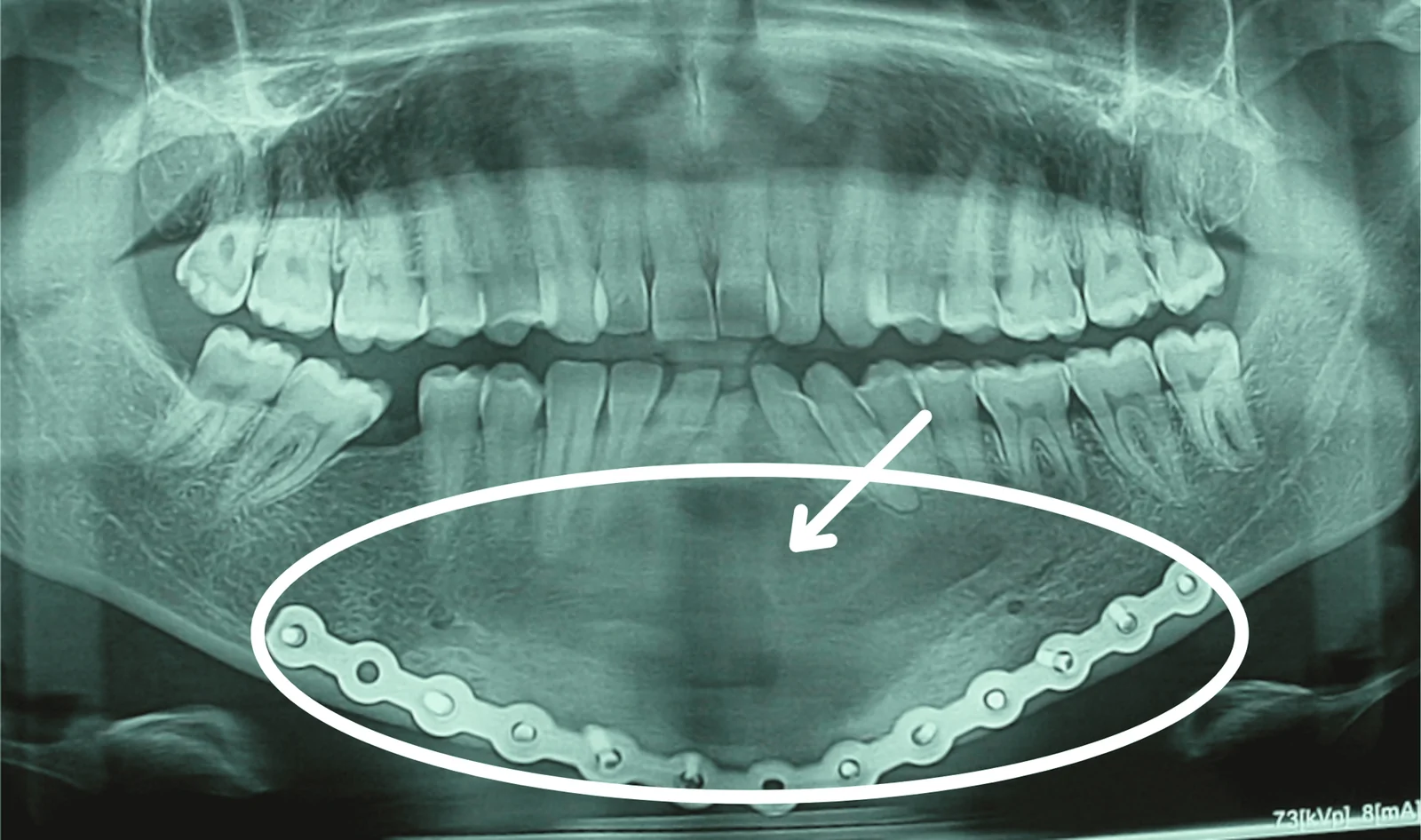
Panoramic X-ray one-year post-op

Recurrence has been reported in between 10% and 15% of CEOT cases; however, the overall prognosis is generally considered favorable according to the literature. Given the potential for recurrence, the patient remains under regular clinical and radiographic follow-up. To date, no evidence of recurrence has been observed. The patient has remained free of clinical and radiographic signs of recurrence during the one-year follow-up period (Figure 9). Given the reported recurrence rate of CEOT and the potential for late recurrence, the patient will continue to undergo annual clinical and radiographic follow-up examinations as part of a long-term surveillance protocol [8].

## Discussion

Nearly half of all reported cases of CEOT have been associated with an impacted tooth or with tooth displacement secondary to tumor growth [4]. This fact has led to controversy regarding the origin of the CEOT.

The recurrence rate of CEOT is reported to be approximately 10%-15%. Malignant transformation is considered extremely rare as well [4]. Displacement of adjacent teeth is a frequent clinical feature of CEOT and may represent the earliest presenting symptom. Notably, this displacement generally occurs without associated tooth loosening or mobility [9]. These findings are consistent with those observed in the present case, where all adjacent teeth demonstrated displacement. Notably, the roots were displaced lingually, while pulp vitality was preserved in all involved teeth.

Originally, Pindborg suggested that CEOT originates from the reduced enamel epithelium associated with an unerupted tooth [10]. Subsequently, Chaudhry et al. highlighted that the tumor cells exhibited morphological features characteristic of squamous epithelium and suggested that, in their case, the neoplastic cells originated from the reduced enamel epithelium associated with the adjacent unerupted tooth [11]. Nevertheless, the growing number of documented intraosseous CEOT cases lacking an association with an unerupted tooth, together with reports of peripheral CEOT, has challenged the notion that the reduced enamel epithelium is the sole tissue of origin. Consequently, alternative sources have been proposed in explaining the histogenesis of this neoplasm [12]. The existence of peripheral variants of CEOT has led to the suggestion that the neoplasm may originate from remnants of the dental lamina or from basal cells of the oral epithelium [4].

As dental practitioners, it is our responsibility to systematically assess any pathological findings within the oral cavity. This evaluation should include precise documentation of the lesion’s location, associated clinical signs and symptoms, and a well-reasoned differential diagnosis [13]. In selected cases, adjunctive diagnostic procedures such as fine-needle aspiration (cyto-punction) or a preoperative biopsy may be performed to further characterize the lesion. Based on these findings, an informed decision can then be made regarding the need for referral to an oral and maxillofacial surgeon for definitive management.

The differential diagnosis in the present case was broad, given that several lesions may mimic the clinical and radiographic appearance of conventional intraosseous CEOT. Specifically, a well-circumscribed mixed radiolucent-radiopaque lesion associated with an unerupted tooth may be observed in various odontogenic and fibro-osseous pathologies. Accordingly, diagnostic considerations included calcifying odontogenic cyst (calcifying cystic odontogenic tumor), adenomatoid odontogenic tumor, ameloblastoma, developing odontome, focal cemento-osseous dysplasia, ossifying fibroma, osteoblastoma, and cementoblastoma.

Adenomatoid odontogenic tumor predominantly affects young adults and demonstrates a female tendency, with the maxilla being the most frequently involved site. Radiographically, it can often be distinguished from CEOT by the presence of scattered radiopaque flecks, commonly referred to as “snowflake” calcifications. Such findings were not evident in the radiographic examination of the present case [8].

Even though both ameloblastomas and CEOTs present as expansile tumors, CEOTs are often radiologically distinguishable due to the presence of intralesional calcifications, as is the case with our patient [8]. Thus, CEOTs produce a mixed radiopaque-radiolucent pattern, in contrast to the typical multilocular soap-bubble, radiolucent appearance of ameloblastoma. Clinically, ameloblastomas are more aggressive in comparison to the typical CEOT, often causing root resorption rather than displacement [14].

Although both lesions often present as mixed radiolucent-radiopaque jaw lesions, ossifying fibromas are characterized by a well-circumscribed, centrifugally expanding lesion with corticated borders, in contrast to CEOT, which demonstrates a more irregular, potentially infiltrative pattern with characteristic calcifications and frequent association with impacted teeth [15].

As mentioned, several entities may be considered in the differential diagnosis of such a lesion. Therefore, a comprehensive clinical and radiological evaluation is essential, maintaining a broad diagnostic perspective. Definitive diagnosis ultimately relies on histopathological examination, which, in the present case, confirmed the lesion as a CEOT.

According to the latest WHO Classification of Head and Neck Tumours, three histological variants of CEOT are currently recognized: clear cell CEOT, cystic/microcystic CEOT, and non-calcifying/Langerhans cell-rich CEOT [3].

Abrams and Howell were the first to document the presence of clear cells in CEOT in 1967, thereby identifying a distinct histopathological feature of the tumor [4]. The majority of clear cell CEOT are intraosseous lesions and are commonly found in the mandible [16]. Unlike conventional CEOT, there is a female predilection, and only 33% of cases are associated with unerupted teeth [16]. The literature has proven that clear cell variants of CEOT exhibit a more aggressive clinical behavior compared with conventional forms, with a higher propensity for cortical bone perforation and a greater tendency for recurrence [16].

Langerhans cell-rich variant of CEOT typically presents as a radiolucent lesion on radiographic examination and is therefore frequently misdiagnosed as an odontogenic cyst. The absence of radiopaque foci is thought to be attributable to the immature nature of the lesion, in which calcification has not yet developed to a detectable extent [16]. These lesions contain abundant Langerhans cells, specialized antigen-presenting immune cells that are normally present within the oral epithelium and have also been identified in small numbers in conventional CEOTs. When present in significant quantities, these Langerhans cell-rich lesions are regarded as a distinct histopathological variant of CEOT [17]. It was stated by Kaushal et al. that “The non-calcified variant of CEOT behaves more aggressively than calcified CEOTs” [18]. Nevertheless, this observation differs from earlier studies proposing that the majority of non-calcifying CEOTs exhibit a prominent population of Langerhans cells, which may be indicative of a less aggressive lesion [17]. Additional investigations involving non-calcifying CEOTs, with and without Langerhans cell infiltration, are warranted to elucidate the relationship between Langerhans cell presence and tumor behavior.

More recently, several reports have described cystic and microcystic variants of CEOT. The first documented case involved a large cystic lesion in a 15-year-old male, where the cystic lining exhibited histopathological features consistent with CEOT [19]. The lesion was managed by enucleation. Subsequent reports have described similar presentations, supporting the existence of a cystic variant. In addition, a microcystic form has been identified, characterized by a pseudo-glandular architectural pattern in association with otherwise typical CEOT histology [20]. The biological behavior and long-term prognosis of these variants remain unclear; however, no cases of recurrence have been reported to date.

The management of CEOT ranges from conservative approaches, such as enucleation and curettage, to more aggressive interventions, including marginal or segmental resection. Guided by the lesion’s clinical presentation and histopathological characteristics, the choice of surgical modality should be decided [5]. Additional factors, including patient age and tumor location, should also be considered when formulating an appropriate treatment plan.

In the present case, the inferior border of the mandible remained intact. Consequently, enucleation of the lesion followed by curettage was selected as the treatment of choice. Following lesion excision, the residual buccal cortical bone, together with a reconstruction plate, was utilized to restore mandibular continuity and reconstruct the resulting defect.

## Conclusions

This case highlights a rare presentation of CEOT, occurring at an unusual anterior mandibular location and in association with odontoma-like calcified structures. Such a combination of clinicopathological features has been infrequently reported in the literature. The clinical and radiographic findings were not pathognomonic and overlapped with those of several odontogenic lesions, underscoring the importance of correlating clinical, radiographic, and histopathological findings to establish a definitive diagnosis and formulate an appropriate treatment plan.

Given the rarity of CEOT associated with odontogenic calcified structures, this report contributes to the existing body of knowledge and may aid in further understanding the clinical presentation and management of this uncommon neoplasm. In the present case, a conservative surgical approach resulted in favorable short-term outcomes, with preservation of the teeth occupying their normal position within the dental arch and maintenance of pulp vitality throughout the follow-up period. Although no evidence of recurrence was observed during the one-year follow-up period, continued long-term surveillance remains necessary due to the potential for late recurrence. This case adds to the limited evidence supporting the feasibility of conservative management in carefully selected cases of CEOT.
